# Real Estate Markets and Lending: Does Local Growth Fuel Risk?

**DOI:** 10.1007/s10693-021-00358-9

**Published:** 2021-09-03

**Authors:** Maximilian Zurek

**Affiliations:** University of Bayreuth, 95440 Bayreuth, Germany

**Keywords:** Lending risk, Regional banks, Collateral, Real estate markets, G21, G32, G11, R31

## Abstract

Real estate price growth affects credit risk for several reasons: it provides input for economic forecasts as it’s closely tied to economic growth; when used as collateral by banks, rising real estate prices may decrease both expected and actual losses; and banks may become less risk averse in lending practices in the presence of rising property prices. Therefore, we analyze these effects on loan portfolios’ estimated and realized risks on a local level. Using data of 390 German savings banks, however, we find that real estate prices have little or no impact on savings banks’ credit portfolio risk or risk precautions.

## Introduction

Real estate markets and investments in real estate have gained increased attention in the aftermath of the financial crisis 2007–2008. Among other reasons, the steady lowering of interest rates has made real estate investments increasingly attractive, as they are not only highly leveraged but are also frequently considered safe investments. This has led to increased real estate prices in Germany, in a few cities in particular (Siemsen and Vilsmeier 2017). Due to the high price and ubiquity of real estate, as well as its economic relevance, property markets cannot work without proper loan markets. In the worst case, this relationship can lead to assigning an increasing number of loans to riskier borrowers who collateralize their debt using real estate.

In order to deter borrowers from defaulting, banks demand collateral (Stiglitz and Weiss 1981), with real estate being the most commonly used collateral device in lending (Niinimäki 2009). Pledging more collateral may be used as signal of lower borrower risk (Agarwal et al. 2015), while demanding collateral may be an indicator of *lazy banks* in the spirit of Manove et al. (2001). Collateral thus has a high potential for inducing banks to issue loans to risky borrowers. Banks not only consider collateral-pledging borrowers to be less risky per se, but may also perform less monitoring when loans are backed by properties. This tends to act as an accelerant on the relationship between property and loan markets: When loans are collateralized with real estate, banks could avoid losses when property prices rise; but if they drop, loan losses are more severe as market values of recoveries are below the exposure at default (EAD) (Niinimäki 2009). Lower capital reserves held by banks with large real estate portfolios (Blasko and Sinkey 2006) could exacerbate the problem. Furthermore, borrowers whose loans were overcollateralized at the beginning may have an incentive to default if the price of the pledged real estate drops below the outstanding amount of credit (Herring and Wachter 1999).

Banks can also be suborned to use current or past real estate prices as indicators for current and future economic development or future real estate prices. Banks expecting high growth of real estate prices in the near future might be willing to accept more high-risk borrowers whose loans are collateralized by real estate.

Taking both of these arguments into consideration, banks anticipate rising trends of real estate values by observing current prices, and therefore may be willing to lend to risky borrowers today in the expectation that the same borrowers will be wealthier in the future, decreasing their default risk via incentives and their expected loss given default (s. Landvoigt 2017).

**Fig. 1:**
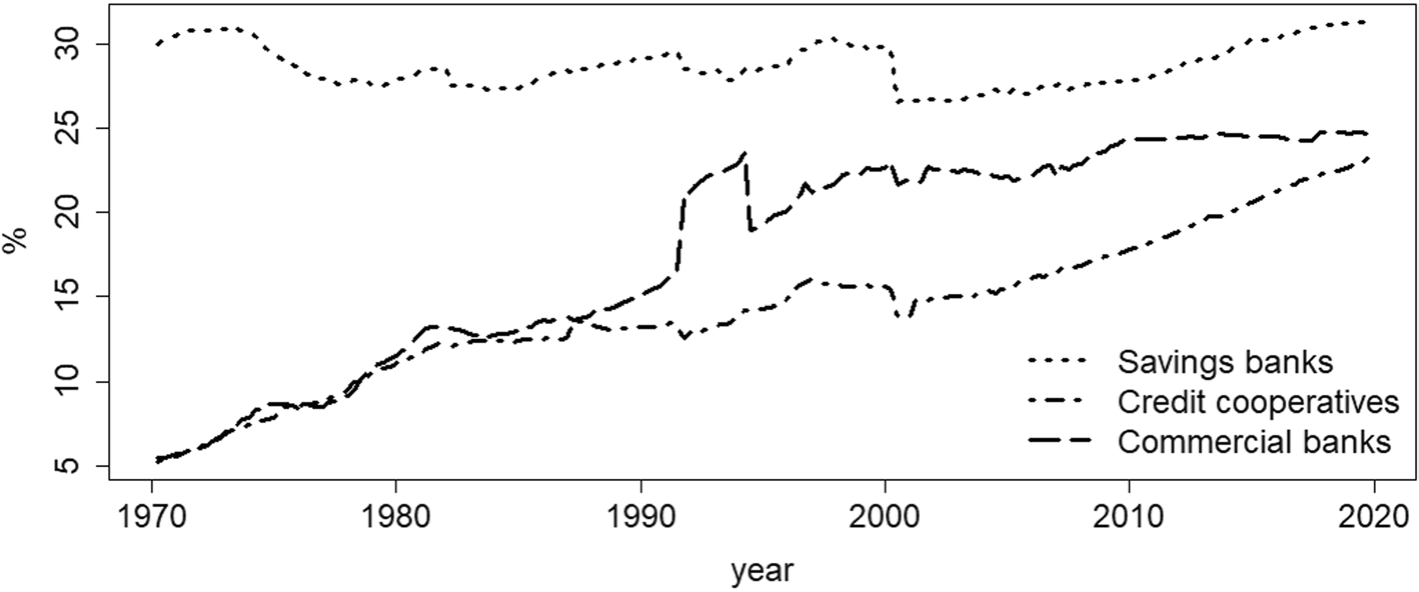
Shares of all housing loans made to German firms and households across bank types. Savings banks’ and credit cooperatives’ business areas are mostly restricted to a few rural and/or urban districts. Data source: Deutsche Bundesbank

**Fig. 2:**
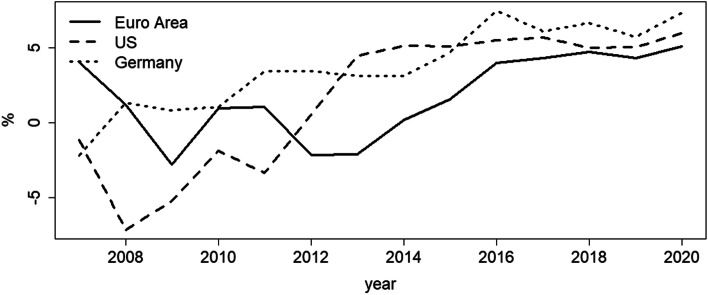
Annual real estate price growth rates of Germany, the U.S. and the Euro Area as of April 2021. Data sources. European Central Bank and U.S. Federal Housing Finance Agency

These issues could be even more pronounced in the case of banks that are regionally constrained and depend on the economic well-being of their surrounding business area. If banks additionally face limitations on their investment policies, they may develop an even stronger dependency on real estate price development. This is, indeed, the case for German savings banks; because they are heavily engaged in real estate related lending (see Fig. 1), they are particularly vulnerable to taking on additional risks when local real estate prices are high. As can be seen from Fig. 1, savings banks have been originating one third of housing loans for over 40 years, which underpins their high relevance for the German real estate market. Including also regionally based Credit cooperatives, about half of German housing loans are originated by local banks. The issue therefore is closely linked to locally based banks and their connection to local lending markets, which has been a competitive advantage for many years. Yet, with the number of branches shrinking and the reduction of personal contact which has been fueled by the COVID-19 pandemic, this advantage could parish. Additionally, real estate prices have soared lately (see Fig. 2), which offers such banks additional opportunities in lending. Therefore, the combination of loss of informational advantage and seemingly increasing profits and lower risk in real estate lending could induce regional banks to switch their lending strategies towards more transaction-based lending. Consequently, real estate price growth, indicating collateral value growth, could affect loan portfolio risk strongly. Thus far, however, micro-evidence on real estate’s impact on risk taking in lending has been scarce.

Therefore, we focus on whether local real estate price growth affects savings banks’ loan portfolio risk. We suggest that strong local real estate price growth could induce banks to be over-optimistic and hence underestimate loan risks. Analyzing this, we use micro level data of 390 German savings banks from 2011 to 2018 with Blundell-Bond-estimators being the method in use. However, we find no robust evidence that savings banks’ loan portfolio risk is driven mainly by real estate price growth or expectations on real estate prices. Results suggest, rather, that savings banks’ loan portfolio risk is affected by bank-specific variables and overall regional and national economic environment. That is, there is a direct link between loan portfolio and local economic conditions, and only an indirect channel via housing prices.

The paper proceeds as follows: In section two, we review the literature on the topic and present the hypotheses that will be tested. Here, we differentiate between the potential effects of real estate price growth on lending and risk-taking behavior of banks. Section three presents the data and discusses the characteristics of German savings banks. Section four presents the results of the empirical investigation, which comprises an analysis of the effects of real estate and loan growth and a second part analyzing the impact of real estate on the risk of banks’ loan portfolio with micro data from German savings banks using dynamic panel data methods. Section five presents conclusions and implications of the study.

## Literature review

The causal relationships between real estate price growth, lending and risk are complex. Our aim is to disentangle these interrelations by identifying four mechanisms that explain how the growth in property prices affect lending behavior.

First, higher house prices not only require higher loan nominal amounts, but owners’ property values also increase. This enables lenders with real estate collateral to obtain higher loan amounts. If banks believe that this growth is sustainable, they will increase their lending. If they do not, price growth has no effect on lending volumes, which slows down real estate price growth. Research on this topic already has been conducted with data on national levels (e.g. Gerlach and Peng 2005), while analyses on local levels (e.g. Favara and Imbs, 2015; Defusco 2018) that take spatial heterogeneities within countries into account when it comes to lending, have been scarce. Yet, due to the heterogeneity of real estate and differences in banks’ lending behaviors (national vs. international vs. local lending), analyses on a large-scaled geographic area could lead to misleading results when it comes to understanding lending practices of regionally based banks. As business areas of those banks are geographically limited, they cannot smooth negative real estate price developments enlarging their business area to include regions with positive real estate price growth. To the best of our knowledge, this issue has been neglected so far when it comes to analyzing regional banks.

The consequences of lending with overvalued collateral have been discussed by e.g. Herring and Wachter (1999) and Siemsen and Vilsmeier (2017). Additional empirical evidence has been provided by Koetter and Poghosyan (2010), who analyze the impact of deviations of real estate prices from fundamental values on banking stability.

Second, high real estate prices might additionally reduce banks’ monitoring efforts and the perceived riskiness of a loan. This can happen either when real estate price growth is used as a predictor for future economic performance or when properties are used as collateral and expected price growth counteracts expected losses. Bester (1985) argues that borrowers with high risk prefer loans with low collateral requirements and are willing to accept higher loan rates. Thus, collateral can offer insights into borrowers’ analysis of risk. Real estate, which per se reduces risk compared to uncollateralized loans, can act as a signal of high-quality borrowers. To the best of our knowledge, empirical evidence on the impact of local real estate prices on lending risk is scarce, yet theoretical analyses have been published, e.g. by Niinimäki (2009) and Bian and Liu (2018).

Third, higher collateral values reduce losses given default (LGD) *ex post*, which is directly linked to ex post risk. Anticipating lower LGDs, lenders may be induced to lend to riskier borrowers, which in turn leads to an ex ante constant risk but an ex post higher risk, i.e. more realized losses. Prior empirical research mainly has focused on other effects on lending, e.g. GDP (Salas and Saurina 2002), unemployment rates (Balasubramanyan et al. 2017) or interest rates (Delis and Kouretas 2011) or issues that are directly attributable to a single loan such as collateral (e.g. Berger and Udell 1990).

Fourth, expected losses are not only based on current information, but also on expectations regarding real estate price growth. Real estate prices are publicly observable, which is not the case for other local indicators of economic performance such as GDP or figures on unemployment. Therefore, banks might base their expectations on future economic and real estate price growth on current and past property prices. Suspecting economic growth, banks might be willing to assign risky loans as they expect borrowers’ solvency to increase on average. If banks expect continued real estate price growth, their expected losses from collateralized losses will decrease. Risky lending, therefore, might increase, potentially creating a large gap between ex post and ex ante risk measures.

### Real estate prices and loan volumes

As Gerlach and Peng (2005) point out, the relationship between loans and real estate prices is evident in several aspects. With real estate frequently serving as collateral, higher housing prices enable borrowers to apply for higher credit amounts. A number of studies have found that financially constrained firms increase their borrowing if the value of their collateral increases (e.g. Agarwal et al. 2015; Cvijanovic 2014; Dougal et al. 2015). Landvoigt (2017) found that households will increase their leverage if real estate prices have increased. Similarly, Defusco (2018) suggested that households will try to smooth their consumption if the values of their homes increase, allowing them to post higher collateral (Koetter and Poghosyan 2010), and thereby reducing LGDs. Furthermore, increases of real estate prices generate profits for borrowers that improve their ability to repay (Zhang et al. 2018).

Additionally, banks’ own real estate assets increase in value and charge-offs of loans decrease with increasing property prices (Herring and Wachter 1999). Leaving other aspects constant, this increase in a bank’s wealth and the lower expected losses strengthens bank capacity to extend of credit. Contrarily, lower credit constraints due to a possible substitution of monitoring with collateral could fuel demand for mortgage or other real estate related loans.

Empirical evidence on the two-way causality between property prices and lending volumes is mixed. According to Gerlach and Peng (2005), banks increased their mortgage lending in Hong Kong after increased competition due to deregulation of the banking industry. The authors further found evidence that extended lending did not have an impact on property prices, but that the causality ran in the other direction. In contrast, Favara and Imbs (2015) found that banking branch deregulation led to a greater volume and higher values of loans, which caused real estate prices to rise.

Although competition and low interest rates fuel extension of credit and could cause higher real estate prices, real estate price growth per se allows for higher collateral amounts. Therefore, we suggest that:

- *H1: Savings banks’ loan volumes grow with local real estate prices.*

Existing studies for the most part have analyzed data on single entities (countries) using time-series techniques. Yet, real estate prices are highly heterogeneous between regions, hence aggregating loan and real estate data on a national level could miss a number of insights. Investigating data from a cross-section of spatial entities could provide additional understanding of the market power of banks, bank-specific loan growth in preceding periods and dependency on local economic development along with house price growth. Stable property price growth has special relevance, as non-sustainable price increases due to deviations from fundamental values can threaten banks’ solvency if their estimation of expected losses is based on current market prices that could be corrected in the future when loans are due. As a consequence, otherwise risky loans have similar expected payments as safe loans, such that loan loss provisions can hardly be correctly determined.

The impact of a strong correction of house prices can be significant for the German financial sector. According to Siemsen and Vilsmeier (2017), a drop in housing prices can lead to losses of several billion Euros, considering only less significant institutions (LSIs). Likewise, Koetter and Poghosyan (2010) found that banks located in areas with high deviations in house prices from their fundamental value have a higher probability of being distressed. Yet, a quick adjustment to fundamental values is no simple matter in real estate markets given that investors generally do not have the possibility of going short. For this reason, real estate markets are considered to be driven by optimists (Herring and Wachter 1999).

To detect some exuberance of real estate prices that might threaten financial stability, the deviation from real estates’ fundamental value is often considered an appropriate measure, as opposed to observed prices (Koetter and Poghosyan 2010). Deviations from fundamental values are more easily noted in smaller entities where this information is readily observable. Because savings banks are very familiar with local markets, they are in a better position to recognize exaggerated prices and thus reject loan applications with offers of over-priced collateral. Instead, they might increase efforts to monitor local markets, with the latter decreasing overall loan volume due to fixed input factors in the short term, thus:

- *H2: If local house price increases are not fundamentally driven, savings banks will decrease their lending.*

### Ex ante risk: economic expectations, collateral, and monitoring

If exaggerated property prices do not result in an extension of credit volumes, loans might only take place when there is an increase of real estate prices and a simultaneous reduction of risks, i.e. decreasing LGDs (Koetter and Poghosyan 2010). Due to low LGDs, banks’ willingness to lend collateralized real estate loans is high (Zhang et al. 2018). This is a consequence of the ability to separate the borrower’s risk from the loan’s risk: a risky borrower could obtain credit if pledging a collateral whose value of recourse exceeds the loan amount (Berger and Udell 1990). Yet, because risky borrowers understand their own lending quality, they will tend to avoid pledging collateral. From a lender’s point of view, this collateral is the most valuable.^1^ In turn, borrowers with low default risk will prefer higher collateral over higher interest rates (Besanko and Thakor 1987). Yet, as observably risky borrowers face stronger demands to provide collateral for a loan, the higher demand for collateral suggests that the probability of default increases with collateralization (Inderst and Mueller 2006). Consistent with the work of Besanko and Thakor (1987) and Niinimäki (2009), Agarwal et al. (2015) found that, when interpreting upfront payments of mortgage loans as collateral, it was younger borrowers in particular who, in spite of having a lower score and lower income, made lower upfront payments on average.

As an alternative to demanding collateral, banks could thoroughly screen and monitor their borrowers, even though this is more time-consuming and costly. Manove et al. (2001) suggested that banks acting in perfect competition preferred to use collateral in lending than screening because it was less costly. The cost efficiency of substituting screening with collateral is even higher for low quality borrowers (Keys et al. 2010).^2^ Positive expectations with respect to local real estate prices make collateral even cheaper compared to screening, resulting in a stronger reliance on collateral in areas with real estate prices forecast to increase.

Screening a borrower using all available information when lending leads to an expected loss, which is the foundation of ex ante risk. Furthermore, write-offs or non-performing loans (NPLs) capture the realized risk of a loan portfolio, i.e. ex post risk (Berger and Udell 1990). We argue that real estate prices have an impact on ex ante risk via expectations on a future willingness to pay and values of collateral. If banks observe current real estate price growth, they are able to predict rising prices in the future. Hott (2011), for example, argued that banks might prefer to stick to momentum forecasts than fundamental real estate values, as they are well diversified, having only minimal risk exposure toward fundamental factors. He also found that banks’ myopic strategies did not take real estate cycles into account. Therefore, banks could consider loans less risky in the future as their collaterals increase, accepting higher risks at the present. This effect is even more pronounced on a local level, as real estate prices vary across regions.

Additionally, as a result of an increase of property prices, the wealth of risky borrowers increases and former collateral barriers are no longer considered a deterrent. We expect these effects to be stronger for locally-based savings banks. Thus, the ex ante risk of loans will, on average, decrease.

- *H3: Real estate price growth reduces ex ante risk.*

Expectations regarding the future economic condition of a household represent an important aspect of the estimation of a loan’s risk. As real estate price increases are caused by various economic factors (Gerlach and Peng 2005), property prices can serve as indicators for overall local economic growth. Compared to other economic variables (e.g. GDP or unemployment rates), real estate prices are observable and indicate the wealth of a region. Therefore, higher household incomes and resulting higher capacity to repay loans could result in growth in local housing prices. According to Hott (2011), banks’ optimism concerning the wealth of a household has a significant impact on real estate prices and can lead to circular effects when it comes to lending. If projecting recent price growth of real estate markets to future prices is not sustainable (Herring and Wachter 1999), worrisome overvaluation would be a consequence and either ex ante risk and/or realized risk will be higher.

### Ex post risk: default and realization of real estate collateral

As Hott (2011) found that lending tightens in response to defaults rather than in anticipation of them, ex ante risk measures could be erroneous. Alterations of economic conditions or ratings during the lifetime of a loan are, instead, reflected by ex post risk measures. These include real estate price growth, which could have a significant contribution to the performance of loans and especially the ex post risk of a loan portfolio. This could happen either by a *collateralization effect*, i.e. by a reduction of losses given defaults (similar to Koetter and Poghosyan 2010) or by an *incentive effect*, i.e. by increasing borrowers’ incentives not to default as losses would increase with real estate prices.

Borrowers’ incentives not to default are higher when the value of their collateralized property increases or price increases are expected. Additionally, the possibility of securing collateral reduces agency costs and information asymmetries, as well as alleviates the funding of borrowers (Cvijanovic 2014). Here, again, we stress the prevalence of provision of collateral by highly creditworthy lenders; As collateral commonly has a higher value for borrowers than for banks and as borrowers may be limited in their use of the pledged asset, pledging collateral can be regarded as costly for the borrower (Agarwal et al. 2015; Coco 2000). Different costs may also arise with the use of collateral, depending on whether *outside* or *inside* collateral is in use (for differentiation, s. Niinimäki 2009). Pledging outside collateral is costly for the borrower according to Bester (1985), whereas for Besanko and Thakor (1987), the lender incurs the costs of collateral. Inside collateral is explicitly without costs for the borrower (Niinimäki 2009).

Turning to the effect of collateralization, banks incur lower ex post risk if prices of real estate collateral increase, given that larger fractions of defaulted loans can be covered. This reduction of realized losses is observable in banks’ charge-offs of loans. Yet, collateral does not decrease a loan’s risk per se. Berger and Udell (1990) found that loans with fixed interest rates and collateral had below average performance, which the authors took as evidence that securing collateral was insufficient to eliminate a loan’s risk.

Furthermore, the on average higher risk premia and higher charge-offs for banks holding more real estate collateralized loans, suggest that loan risk cannot be fully covered by collateral and that these loans bear some degree of high risk. Similarly, Blasko and Sinkey (2006) found that banks that are highly engaged in real estate lending over several years have lower net loan losses. This is partly confirmed by Zhang et al. (2018), who found a negative relationship between local growth of real estate investments and NPLs, i.e. ex post risk is decreased, possibly due to the higher collateralization of loans. The authors also found that a reduction in the level of real estate market activity renders banks that are strongly engaged in real estate lending unstable. Salas and Saurina (2002) argued that the impact of GDP growth on Spanish savings banks’ ratio of problem loans is weaker than that of commercial banks, which have more customers dependent on business cycles. Thus, savings banks’ lending success may be attributed to local economic factors that are more independent of national business cycles. As real estate prices are strongly linked with the local economy and economic cycles, our results should be similar to those of Salas and Saurina (2002).

We expect past real estate prices or expectations of real estate price growth to decrease ex post risk via the collateralization and the incentive channel:

- *H4: Real estate prices decrease ex post risk.*

### Default forecasts and over-optimistic expectations of real estate prices

Not only are real estate investments commonly regarded as having little risk, but local lending is also perceived as relatively low risk due to spatial proximity, or ‘home bias’ in lending. Both factors contribute to the potential underestimation of the risk of lending with real estate collateral in a local setting. Therefore, expectations of risk and future property prices as determinants of LGDs could be mediating factors between real estate prices and lending behavior.

Several studies deal with potential links between current real estate prices and banks’ expectations of future risks. Hott (2011) argues that positive income shocks may have an impact on price expectations, leading to current price increases. As banks base their expectations on current prices, expected prices then increase and continue the feedback process. According to Zhang et al. (2018), the threat to financial systems posed by falling real estate investments has the potential to be severe if it becomes a correction to the housing market. Recent experiences in house price development influence not only lenders’ expectations of future prices, but also those of borrowers, who are therefore able to make higher upfront payments for mortgage loans (Agarwal et al. 2015) or apply for loans, even when unable to signal low risk and receive a favorable contract. Thus, there might not only be a higher offering of credit, but also demand from risky borrowers.

Indeed, in an analysis of data on U.S. homeowners, Defusco (2018) found that loans obtained by extracting equity, i.e. using the increase in property value to obtain additional loans, are riskier than comparable loans, with the risk being measured using foreclosures.

In the case of expected increasing real estate prices, banks attempting to maintain a constant aggregate net present value (NPV) for loans are willing to grant loans to borrowers with higher probabilities of default. Banks will continue lending to risky borrowers as long as the annual increase in the overall default probability is less than the expected annual growth of collateral value. This tendency may be exacerbated when relying excessively on collateral as opposed to screening; the deteriorating survival probabilities of loans cannot be observed directly, while properties can be appraised on an ongoing basis. Given the potential for unanticipated losses (see section 2.2), engaging in riskier lending behavior when relying on estimates of future collateral values may have considerable impact on banks.

Therefore, on average, a lower ex post risk than suggested by ex ante risk measures occurs if banks have not anticipated the actual positive real estate price growth. When real estate price growth is weaker than expected, the ex post risk will be higher than suggested by the ex ante risk. In both scenarios, the gap between the two measures widens with recent real estate price growth.

We consider this gap between loan risk provisions and realized loan risks as a potential scenario for German savings banks. Legal limitations on their business activities and their embeddedness in local economies may lead to overconfidence in forecasting local real estate prices. As political impacts concerning property price and economic growth may reinforce local forecasting (Illueca et al. 2014), we formulate our final hypothesis:

- *H5: Higher recent real estate price growth weakens banks’ risk forecasting ability.*

### German savings banks

Savings banks are a major constituent of the German banking system and are highly relevant for business financing and retail customers. Their loan volumes to non-monetary financial institutions (MFIs) grew by almost 24% from the beginning of 2011 to the end of 2017, leading to a loan volume toward non-MFIs of 951 billion Euros (Data: Deutsche Bundesbank). Loans toward non-MFIs originated by savings banks constituted about 24% of all loans originated in Germany toward non-MFIs by the end of 2018, starting from 19% during the onset of the financial crisis of 2007/2008 (Data: Deutsche Bundesbank). This underlines the high relevance of savings banks within the German financial system. Aside from being highly relevant in Germany, savings banks are a commonly used representative for locally based banks: The focus of their business is deposit-lending; investment banking activities play a negligible role for most savings banks. This strong similarity of basic investment policies is an additional advantage for the analysis, as there should not be systematic variations of risk-taking (Conrad et al. 2014). Furthermore, their dense network of branches allows to draw consequences from the results for regional financial institutions where – due to the connection towards the region - funding and lending practices are highly correlated.

Koetter and Poghosyan (2010) found that savings banks have an even lower probability of becoming distressed than small-sized and regionally-based cooperative banks, which may be a partial reflection of their aversion to business risk.

German savings banks are also restricted geographically in terms of their area of operation and they are present throughout the whole country. These business areas typically coincide with urban or rural districts or cities (similar to Metropolitan Statistical Areas). Because these banks are particularly sensitive to local economic variables (e.g. Reichling and Schulze 2018), they are convenient for analyses that may include business areas and local factors (Conrad 2008).

Salas and Saurina (2002) found that bank level characteristics, such as market power and local indebtedness of borrowers, have a high explanatory power for the growth of problem loans in the case of Spanish savings banks. Thus, notable differences between results of analyses on an aggregate and individual level could be based on the sensitivity of locally operating banks toward their particular economic environment.

Given savings banks’ mandate to guarantee a supply of funding within their business areas, they are likely to have a higher exposure to riskier borrowers than more transaction-based-lenders.^3^ As local lenders, they are likely to demand higher collateral and lower interest rates for loans than transaction-based lenders (Inderst and Mueller 2006).^4^ Savings banks are also likely to be more dependent on mortgages due to their limited investment opportunities, rendering them especially vulnerable to changes in local real estate markets.

## Data

Using micro-level data of regions with varying real estate growth permits us to analyze whether the relationship between property investment and NPLs is sensitive to property cycles (Zhang et al. 2018). While there are advantages to using land prices to gauge real estate price development (e.g. Cvijanovic 2014), real estate data is available only on a local level through recorded transactions. The data, including number of transactions, size, prices and location of sold properties, may also vary across time and between jurisdictions. County-level real estate data were obtained from *empirica ag*’s quarterly database, using offered buy and rent prices of house and condo prices with hedonical adjustments. Hedonic house price indices use data from actual transactions and offers and therefore include a variety of information sources that cover a large portion of local real estate markets. Their correction for individual property characteristics overcomes bias in the data as a result of low transaction volumes and due to different qualities of real estate transactions. To have greater comparability and ability to calculate price-rent ratios, we use prices and rents for houses and condominiums of all ages.

We collected information on the entities that are included in savings banks’ official jurisdictions from their annual accounting reports, the addresses of their branches, their statutes or other reports published on their homepages. We cross-referenced this information with county data on real estate price growth and brought to a single value by calculating averages.

Unemployment rates within business areas are publicly available from the German Federal Employment Agency.

The core of the empirical investigation uses micro-level data on German savings banks, obtained by Orbis Bank Focus. Bank-level data include data from balance sheets and profit and loss statements, including information on NPLs, loan loss reserves and loan net charge-offs on the portfolio level, which can represent ex ante or ex post risk measures. The unbalanced panel dataset comprises 390 savings banks with observations spanning from 2011 to 2018. Performance differences of loans might occur even when controlling for hard facts due to variations in the use of soft information (e.g. Keys et al. 2010). Because this type of information is frequently used by small local banks when granting loans, it may also affect savings banks, which exhibit some similarities to privately managed banks.

## Empirical investigation

In her empirical investigation, Cvijanovic (2014) assumed that a firm’s real estate assets were located in the same MSA as the firm’s headquarters. Similarly, we assumed that mortgage lending and collateralization only took place in the savings banks’ jurisdictions and in the areas of their supporting agencies. We took into account real estate prices from the counties where the bank has branches or in cities where the banks’ supporting agencies operated. As the exact geographic origins of loans and deposits were not documented, we refrained from spatial weighting methods.

We used dynamic panel data estimation as risk-taking might have some persistence due to long-term relationships with borrowers, competition, and other external circumstances. Delis and Kouretas (2011), using a Blundell-Bond estimator, found that risk-taking is highly persistent for the first lag. Furthermore, savings banks often assign long-term loans, for which the risk transfers from the preceding period. Additionally, there could be autocorrelation in NPL ratios as found by Zhang et al. (2018).

As dynamic panel data estimation implies endogeneity via construction, ordinary least square procedures would produce biased results. Endogeneity can also potentially be found in several explanatory variables, such as efficiency (Conrad et al. 2014), real estate prices, and others. The use of GMM estimators is a common method to overcome the problem of first differencing resulting in a short panel bias (Behr 2003; Flannery and Hankins 2013). To model persistence of ex ante and ex post loan risks and the impact of real estate prices, Arellano-Bond-Estimators (Salas and Saurina 2002) and Blundell-Bond System-GMM are commonly used (Delis and Kouretas 2011; Zhang et al. 2018). The use of the latter is justified by persistence of the explained variable. In such cases, the first differences as employed by the Arellano-Bond estimator are rather weak instruments (Baltagi 2008, p. 160f). As first estimations yielded some high persistence of our measures of ex ante and ex post risk, we employed the System GMM estimator in what follows. We employed Windmeijer’s finite-sample correction (Windmeijer 2005; also used e.g. by Olszak et al. 2018) to guarantee the robustness of the estimation results.

### Estimation and variables

In this section we briefly present the variables used in the following estimations.

Following Salas and Saurina (2002) and Olszak et al. (2018), we used the following estimations to grasp the impact of real estate price growth on loan risk:

$${\displaystyle \begin{array}{c}{Problem}\ {Loan}{{s}}_{{i}{t}}={\upalpha}_1{Problem}\ {{Loans}}_{{i}{t}-1}+\sum\limits_{{k}=1}^3{\upgamma}_{{k}}\ \widehat{{{GCL}}_{{i}{t}-{k}}}+{\updelta}_1{{LTA}}_{{i}{t}}+\\ {}\sum\limits_{{k}=1}^3{\upphi}_{{k}}{{TCAR}}_{{i}{t}-{k}}+{\updelta}_2{{Lerner}}_{{i}{t}}+{\updelta}_3\ {{monitoring}}_{{i}{t}}+{\updelta}_4\ {{CIR}}_{{i}{t}}+{\updelta}_5{{NIM}}_{{i}{t}}+\sum\limits_{{k}=1}^3{\uppi}_{{k}}\ {{profits}}_{{i}{t}-{k}}\\ {}+\sum\limits_{{j}=0}^2{\upbeta}_{{j}+1}\ {Real}\ {Estate}\ {Price}\ {{Growth}}_{{i}{t}-{j}}+{\upeta}_{{i}}+{\upepsilon}_{{i}{t}}\end{array}}$$

Where we varied the lags of the explanatory variables, considering different points of time of ex ante and ex post risk. For the sake of simplicity, bank and business area specific variables are both denoted by i, with real estate price growth as business area variable being measured in different ways. Variable definitions and data sources are displayed in Table 1.

**Table 1:** Variable definitions and data sources

Variable name	Variable Description	Data Source
$\widehat{{branches}}$	Growth of number of banks’ branches	Bank Focus
CIR	Cost-to-Income-Ratio $\left(\frac{{operating}\ {expenses}}{{operating}\ {income}}\right)$	Bank Focus
$\widehat{{condoP}}$	Growth of condo prices (Euro/sqm) within banks’ business area	Empirica AG
$\widehat{{GCL}}$	Growth of Gross Costumer Loans	Bank Focus
GDP	GDP per Employee in thsd. Euros	German Federal Statistical Office
$\widehat{{houseP}}$	Growth of house prices (Euro/sqm) within banks’ business area	Empirica AG
HP Deviation	Deviations from fundamental house prices (based on own calculation)	own calculations
impaired	Impaired loans / Gross costumer loans	Bank Focus
Lerner	Lerner Index for market power (based on own calculation)	own calculations
LLR	Loan loss reserves / Gross customer loans & advances	Bank Focus
LLRIMP	Loan loss reserves / Impaired loans	Bank Focus
loans/TA	Loans/Total Assets	Bank Focus
LTA	Natural logarithm of Total Assets	Bank Focus
monitoring	Monitoring Effort proxied by $\frac{{Number}\ {of}\ {employee}{s}}{{Gross}\ {loans}\ {to}\ {costumer}{s}}$	Bank Focus / own calculations
NCO	Net charge offs / Average gross customer loans & advances	Bank Focus
NIM	Net Interest Margin $\left(\frac{{interest}\ {income}-{interest}\ {expenses}}{{interest}\ {earning}\ {assets}}\right)$	Bank Focus
$\widehat{{population}}$	Growth of population (in %) within banks’ business area	German Federal Statistical Office/own calculations
PopDens	Population Density as inhabitants per square km within banks’ business area	German Federal Statistical Office/own calculations
profits	Profit (loss) before tax	Bank Focus
PRR	Price-to-rent ratio within banks’ business area $\left(\frac{{house}\ {prices}\ {in}\frac{{Euro}}{{sqm}}}{{rents}\ {in}\ \frac{{Euro}}{{sqm}}}\right)$	Empirica AG /own calculations
TCAR	Total Capital Ratio $\left(\frac{{Capital}\ \left({total}\right)}{{Risk}\ {weighted}\ {assets}}\right)$	Bank Focus
unemp	Local unemployment rates within banks’ business area	German Federal Employment Agency

We used several variables as bank specific controls as suggested by Delis and Kouretas (2011). Banks’ equity ratios capture the deliberations between an increase in risk necessary for capital requirements. Furthermore, equity ratio has an impact on banks’ risk taking itself. To take this into account, we included banks’ capital ratios (*TCAR*_*it*_), similar to Olszak et al. (2018). As Reichling and Schulze (2018) found German savings banks located in wealthier regions to be more efficient, we included the cost income ratio (*CIR*_*it*_) to take this into account. We used net interest margins (*NIM*_*it*_) to account for banks’ ability to generate earnings by assigning new loans and their general profitability (s. Blasko and Sinkey 2006).

As Hott (2011) pointed out, past profits affect banks’ optimism regarding future earnings. Hence, lagged profits may impact not only lending volumes, but also the riskiness of loans in several ways. On the one hand, low profits might lead banks to engage in more and/or riskier lending. Contrarily, it could also lead them to reduce risky loans to prevent further losses. Here, *profits*_*it*_ is defined as $\frac{Profits\ and\ losses\ before\ taxe{s}_{it}}{Total\ asset{s}_{it}}$.^5^

We included branch growth as an explanatory variable for loan volume growth ($\widehat{branche{s}_{it}}$) (Illueca et al. 2014). As business areas of savings banks are bindingly defined and rather small (commonly equal to one or two counties), we did not include branch growth in the additional risk estimations as geographical expansions into other market areas are not conducted.

Furthermore, banks with a high portion of real estate lending have higher loan to asset ratios, which may also affect their provisions for loan losses (Blasko and Sinkey 2006). Thus, this ratio was also included (Sinkey and Greenawalt 1991) as *loans*/*TA*_*it*_, calculated as $\frac{Gross\ costumer\ loan{s}_{it}}{Total\ asset{s}_{it}}$.

Because banks’ higher risk taking or higher losses may result from less monitoring, monitoring intensity, proxied by $\frac{Number\ of\ employee{s}_{it}}{Gross\ loans\ to\ costumer{s}_{it}}$ was included (Kick and Prieto 2015). A further issue worth noting is a potential deterioration of monitoring activity (Manove et al. 2001). Thus, monitoring could be endogenous as well, with real estate price growth having a strong impact on it.^6^

Another common explanation of bank risk taking is competition between financial intermediaries (e.g. winner’s curse) (Forssbaeck and Shehzad 2015). For locally-based savings banks, measures of competition take into account several dimensions of local lending and borrowing, including local wealth, the share of county deposits, the number of branches within an area, interest income per branch, etc. Yet, most of this information was either not available at all or had low explanatory power due to a variety of factors, such as different hierarchies of branches of commercial banks. Furthermore, classical measures of market concentration, such as the Herfindahl-Hirschman-Index (HHI) on deposits on a county level, are often not bank-specific variables, but rather locally dependent and their effects can be proxied by county-dummies. Thus, as a common index on a bank-level, we employed a Lerner-Index (Lerner_it_) based on the procedure described in Berger et al. (2009) (see Appendix).

Similarly, the growth rate of gross costumer loan volume ($\widehat{GC{L}_{it}}$) could be an indicator of whether higher loan losses were due to an unequal growth of loan quality and quantity regarding ex post risk. Higher ex ante risk with higher loan growth, however, might indicate a market expansion or confidence in future returns, possibly induced by growing real estate prices.

As savings banks should have similar standards and techniques in lending, loan loss reserves relative to gross costumer loans for each bank and year (*LLR*_*it*_) were used as the measure of ex-ante risk to capture how observed credit risk was priced before actual losses occurred. Ex post risk was gauged using the banks’ impaired loans, divided by gross customer loans (*impaired*_*it*_). This wider definition of ex post risk captures most of the credit that belongs to problem loans in the spirit of Salas and Saurina (2002).

We used three different measures for real estate price growth: Two of them were growth of house prices within counties, matched with savings banks’ business areas ($\widehat{house{P}_{it}}$) as well as growth of condo prices ($\widehat{condo{P}_{it}}$). Sinkey and Greenawalt (1991) found regional economic factors, proxied by dummy variables, to explain only a very small fraction of loan loss variation of banks. The authors concluded that loan loss rates were instead driven by managerial abilities. This stresses the relevance of managers’ perception of real estate markets and their estimates.^7^ To take this into account, we used price-to-rent-ratios on the county level matched with business areas (*PRR*_*it*_). Note that price-to-rent-ratios not only capture potential deviance from fundamental values, but also future expectations considering real estate prices. Price-to-rent-ratios therefore serve as an observation of local market expectations whereas past price developments are *input* data for individual expectations. Thus, *PRR*_*it*_ captured the effects of the incentive channel (i.e. a borrower’s incentive to repay her loan in order not to lose her collateral with expected price growth).

Summary statistics for the dependent and real estate variables can be found in Table 2. The data were not trimmed or corrected for outliers, and the means are in line with those in other studies. For example Balasubramanyan et al. (2017), using US-based data from 1997 to 2011, found that all loan loss reserves represented 1% of total assets on average, while in our study it was 0.844%.

**Table 2:** Summary statistics. Bank specific data comprise 390 German savings banks, house and condo price growth and price-to-rent-ratios are gauged on the level of 401 urban and rural districts. Real estate prices are hedonic price indices. Circumflex denotes growth variables

Variable	Mean	SD	Min	Max	Obs
$\widehat{{Gross}\ {Loans}}$	0.0383	0.0758	−0.2446	1.9493	1729
$\frac{{Loan}\ {Loss}\ {Reserves}}{{Gross}\ {Loans}}$	0.0136	0.0093	0.0001	0.0897	1997
$\frac{{Impaired}\ {Loans}}{{Gross}\ {Loans}}$	0.0263	0.0186	0.0001	0.171	1937
$\widehat{{House\,Prices}}$	0.0463	0.052	−0.1483	0.3145	2723
$\widehat{Condo\, Prices}$	0.0617	0.0766	−0.379	0.657	2730
Price − to − rent − ratio	20.0995	4.1881	6.4313	51.6336	3120

In order to determine the extent to which real estate prices reflected local economic development, we employed growth of unemployment rate in the banks’ business areas ($\widehat{unem{p}_{it}}$). It should be emphasized that the dynamic panel analysis focused on local real estate price development, thus overall national real estate price growth/decline was only considered within year dummies, which also controlled for the effects of low interest rates and a higher stock market turnover. These parameters have a high stake at determining banks’ risk-appetite. Delis and Kouretas (2011) analyzed risk-taking behavior of banks in 16 Euro-zone countries from 2001 to 2008 and found that banks in a setting of low interest rates shifted their business to more risky investments, as well for ex ante risk (captured by $\frac{risk\ assets}{total\ assets}$) as ex post risk ($\frac{NPL}{gross\ loans}$). Furthermore, banks redistributed their assets to more risky and non-standard banking assets in the presence of low interest rates.

### Loan growth

The first estimation is additional micro-evidence to previous studies based on aggregate levels in order to detect regionally-based causal relationships between loan volume and housing price growth. With regard to the following estimations, higher loan volumes or extension of credit in response to increases in real estate prices could forego higher loan risks if good borrowers already have obtained credit without extension of loans.

We included current (yearly) real estate price growth in order to identify correlations (Gerlach and Peng 2005) that may be caused by the stated *current* observability of real estate price growth, as opposed to e.g. GDP growth, and the time interval in years that allows for an impact of current values.^8^ As an additional explanatory variable, we used ex post risk of two previous periods to check whether past negative experiences concerning credits had a negative effect on current loan growth. The results are displayed in Table 3.

**Table 3:** Results of Blundell-Bond-Estimation of growth of gross costumer loans. The estimation uses different lag lengths for level and difference instruments and employs Windmeijer’s robust standard errors. Growth variables are denoted by circumflex

	(1)	(2)	(3)	(4)
Dependent Variable:	$\widehat{{GC}{{L}}_{{it}}}$	$\widehat{{GC}{{L}}_{{it}}}$	$\widehat{{GC}{{L}}_{{it}}}$	$\widehat{{GC}{{L}}_{{it}}}$
$\widehat{{GC}{{L}}_{{it}-1}}$	0.152	0.109	0.153	0.169
impaired_it − 1_	-0.007**	-0.006*	-0.005	-0.006*
impaired_it − 2_	0.001	0.000	0.000	-0.001
loans/TA_it − 1_	-0.474***	-0.459***	-0.475***	-0.459***
loans/TA_it − 2_	0.047	0.029	0.085	0.074
TCAR_it_	-0.004	-0.002	-0.003	-0.006*
TCAR_it − 1_	0.003	0.001	0.001	0.004
Lerner_it_	-0.224	-0.118	-0.131	-0.152
monitoring_it_	-0.314**	-0.337**	-0.289**	-0.289**
CIR_it_	-0.001	0.000	0.000	0.000
NIM_it_	0.017	0.002	-0.015	0.007
profits_it − 1_	5.657*	5.911*	6.534*	5.153**
profits_it − 2_	2.437	2.702	2.584	2.136
$\widehat{{{branches}}_{{it}}}$	-0.047**	-0.042**	-0.047**	-0.045
$\widehat{{{branches}}_{{it}-1}}$	-0.009	-0.011	-0.010	-0.003
$\widehat{{house}{{P}}_{{it}}}$	0.027			0.022
$\widehat{{house}{{P}}_{{it}-1}}$	-0.015			-0.009
PRR_it_		-0.001		
PRR_it − 1_		0.002		
$\widehat{{condo}{{P}}_{{it}}}$			-0.024	
$\widehat{{condo}{{P}}_{{it}-1}}$			-0.034*	
unemp_it_				0.008
unemp_it − 1_				-0.004
N	546	546	546	546
Number of instruments	72	72	72	78
Year dummies	Yes	Yes	Yes	Yes
First order Arellano Bond Test	-3.03***	-2.82***	-2.98***	-3.26***
Second order Arellano Bond Test	-0.1	-0.49	-0.53	-0.14
Hansen Statistic	50.55	43.53	44.82	55.11
*p* value Hansen Statistic	(0.298)	(0.576)	(0.522)	(0.395)

Significance levels are indicated by * *p* < 0.10, ** *p* < 0.05, *** *p* < 0.01

The results indicate that the major drivers of loan growth were losses and impaired loans of the previous period (i.e. recently made experiences in lending), monitoring efforts, the relevance of lending for the bank’s business, and branch growth. None of the real estate price growth variables, nor unemployment growth as a proxy for regional economic development, were statistically significant in any of the estimations.

**Fig. 3:**
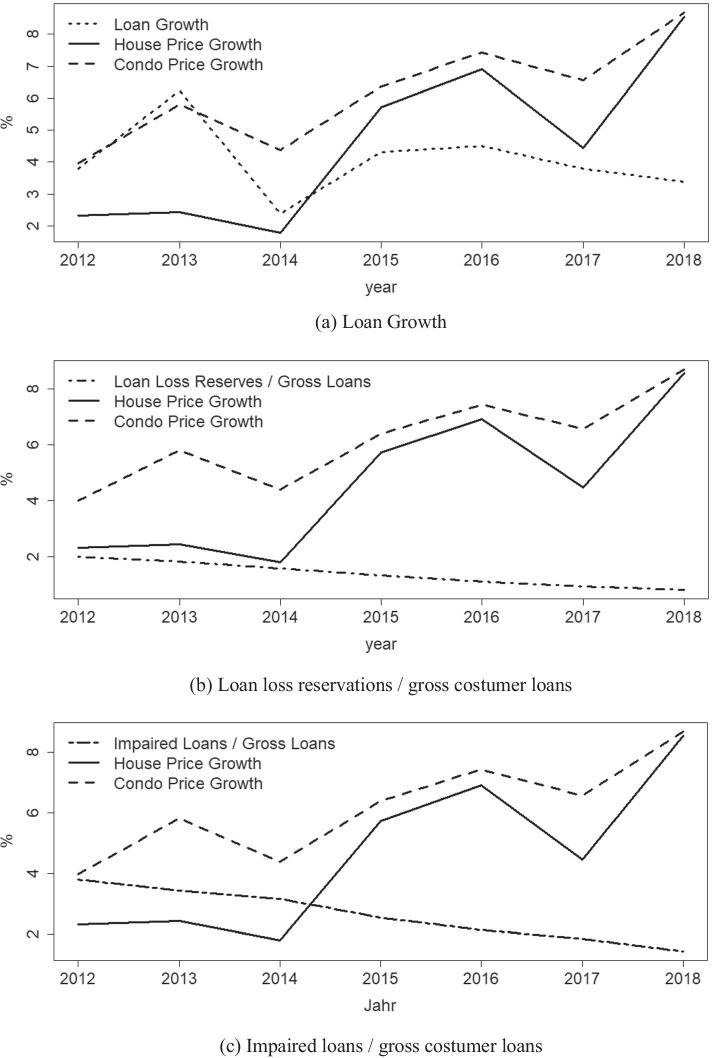
Loan Growth, Loan loss reservations, and impaired loans of German savings banks. Graphic representation of variables of the Micro-Dataset in use together with house and condo price growth; Values represent sample averages. **a** Loan Growth. **b** Loan loss reservations / gross costumer loans. **c** Impaired loans / gross costumer loans

The results disprove the first hypothesis on a local short term (yearly) level. We suspect that savings banks’ reactions to real estate prices were not notable on an overall loan volume level, which could be due to a rather inelastic loan supply. A positive correlation on an aggregate level as graphically suggested by Fig. 3 therefore cannot be confirmed.

The coefficients on price-to-rent-ratio were not significant and changed signs, indicating that market expectations on real estate price growth did not have an impact on loan volumes. We also checked whether the reverse direction of causality would apply to the data and estimated the equation using loan growth as explanatory and house price growth and price-to-rent-ratio as dependent variables (including their lags as right hand side variables). This neither produced significant coefficients nor superior overall results. As loan volume is only available for savings banks, not for the whole county, results of this estimation could be biased.

For testing hypothesis two, the change of loan growth and deviance of prices from the fundamental value, we conducted regressions with the same control variables using price-to-rent-ratio growth, squared price-to-rent-ratios, a dummy, if the current and lagged price-to-rent-ratio exceeds the yearly averaged ratio by more than 10 %, and interaction terms with this lagged dummy variable and house price growth (Table 4). None of these were significant, although this result does not necessarily indicate that savings banks did not react to the exuberance of real estate markets; higher ratios might be justified in certain locations and thus not represent an exaggeration of prices.

**Table 4:** Results of Blundell-Bond-Estimation of ex ante risk. Ex ante risk is measured as$\frac{loan\ loss\ reserve{s}_{it}}{gross\ costumer\ loan{s}_{it}}$. The estimation uses different lag lengths for level and difference instruments and employs Windmeijer’s robust standard errors. Growth variables are denoted by circumflex

	(5)	(6)	(7)	(8)
Dependent variable	*LLR*_*it*_	*LLR*_*it*_	*LLR*_*it*_	*LLR*_*it*_
LLR_it − 1_	0.809***	0.814***	0.796***	0.817 ***
$\widehat{{GC}{{L}}_{{it}}}$	-1.032*	-0.817	-0.994*	-1.143*
$\widehat{{GC}{{L}}_{{it}-1}}$	-0.08	-0.059	-0.073	-0.068
LTA_it_	0.051*	0.052*	0.062**	0.045
TCAR_it − 1_	-0.027	-0.015	-0.012	-0.025
TCAR_it − 2_	0.038*	0.025	0.023	0.033*
Lerner_it_	0.513	0.282	0.838	0.763
monitoring_it_	-0.313	-0.174	-0.310	-0.422
CIR_it_	-0.003	-0.003	0.000	-0.002
NIM_it_	0.041	0.043	-0.050	0.049
profits_it − 1_	-16.470	-14.640	-12.860	-20.377
profits_it − 2_	-8.820	-8.350	-5.458	-13.041
$\widehat{{house}{{P}}_{{it}}}$	-0.722*			-0.436
$\widehat{{house}{{P}}_{{it}-1}}$	-0.070			-0.213
$\widehat{{condo}{{P}}_{{it}}}$		-0.305		
$\widehat{{condo}{{P}}_{{it}-1}}$		-0.075		
PRR_it_			-0.030**	
PRR_it − 1_			0.018	
unemp				-0.094
unemp_it − 1_				0.091
N	1,175	1,176	1,176	1,175
Number of instruments	56	56	56	61
Year dummies	Yes	Yes	Yes	Yes
First order Arellano Bond Test	-4.48***	-4.17***	-4.28***	-4.55***
Second order Arellano Bond Test	1.06	0.78	0.79	1.05
Hansen Statistic	28.97	36.59	31.06	30.63
p value Hansen Statistics	(0.668)	(0.275)	(0.564)	(0.829)

Significance levels are indicated by * *p* < 0.10, ** *p* < 0.05, *** *p* < 0.01

### Ex ante risk

Ex ante risk cannot be determined unambiguously, as higher loan loss provisions can either indicate higher expected loan losses or lower underwriting quality (Dou et al. 2018). Olszak et al. (2018) argue that large banks are more procyclical and more prone to moral hazard due to too-big-to-fail thinking. Hence, we additionally included bank size as an explanatory variable, measured as natural log of total assets (*LTA*_*it*_). Yet, as savings banks are supported publicly and are not excessively large, those problems are not expected to be especially relevant for the estimation.

As can be seen from Table 5, savings banks’ loan loss reserves decreased with current house price growth and price-to-rent ratios, i.e. future market expectations. As stated initially, house prices — in contrast to GDP — can be observed continuously. Thus, current house price growth includes all observations during the year, technically enabling them to have a *simultaneous* impact on end-of-the-year loan loss rates. The effects were not highly significant, which is partly due to the Windmeijer correction. Further lagged house prices did not have a significant impact, which is partially due to the use of lagged loan loss rates, which reflect the explanatory power of the lagged house prices.

**Table 5:** Results of Blundell-Bond-Estimation of ex post risk. Ex post risk is measured by $\frac{impaired\ loan{s}_{it}}{gross\ costumer\ loan{s}_{it}}$. The estimation uses different lag lengths for level and difference instruments and employs Windmeijer’s robust standard errors. Growth variables are denoted by circumflex

	(9)	(10)	(11)	(12)
Dependent variable	impaired_it_	impaired_it_	impaired_it_	impaired_it_
impaired_it − 1_	0.989***	0.979***	0.983***	0.959***
$\widehat{{GC}{{L}}_{{it}-1}}$	0.197*	0.193*	0.220**	0.201*
$\widehat{{GC}{{L}}_{{it}-2}}$	0.301**	0.284**	0.338**	0.276**
TCAR_it − 2_	-0.006	-0.003	-0.003	-0.011
TCAR_it − 3_	0.009	0.015*	0.011	0.011
Lerner_it − 1_	-1.542**	-1.138	-0.984	-1.566*
Lerner_it − 2_	-0.026	-0.040	-0.022	0.013
monitoring_it − 1_	-0.487	-0.752	-0.776	-0.598
monitoring_it − 2_	0.421	0.368	0.534	0.372
CIR_it_	-0.002	-0.001	-0.001	-0.003
CIR_it − 1_	-0.008**	-0.006	-0.006	-0.009**
$\widehat{{house}{{P}}_{{it}-1}}$	-0.189			-0.180
$\widehat{{house}{{P}}_{{it}-2}}$	0.045			0.041
PRR_it − 1_		-0.011		
PRR_it − 2_		-0.002		
$\widehat{{condo}{{P}}_{{it}-1}}$			-0.026	
$\widehat{{condo}{{P}}_{{it}-2}}$			-0.118	
unemp_it − 1_				-0.030*
unemp_it − 2_				0.045
N	793	793	793	793
Number of instruments	54	54	54	61
Year dummies	Yes	Yes	Yes	Yes
First order Arellano Bond Test	-2.38**	-2.40**	-2.38**	-2.37**
Second order Arellano Bond Test	0.76	0.85	1.08	0.62
Hansen Statistic	37.79	37.17	39.78	38.69
p value Hansen Statistic	(0.222)	(0.243)	(0.162)	(0.574)

Significance levels are indicated by * *p* < 0.10, ** *p* < 0.05, *** *p* < 0.01

Although the results of estimation (5) indicate that banks reduced their loan loss provisioning (i.e. ex ante risk) in the face of increasing property prices, the effect was not robust. As can be seen from estimation (8), the coefficient of unemployment growth was stronger in terms of statistical significance. Local house price growth may reflect some degree of overall local economic growth effects, which is supported by the insignificance of condo price growth.

The negative coefficient of the price-to-rent-ratio, indicating lower ex ante risk, was small in economic significance, and the changing sign for the coefficient of the preceding year indicated weak robustness. We will therefore perform a closer analysis of the effects of deviations of real estate prices from the fundamental value in the latter sections.

Additional non-dynamic panel system GMM estimations using $\widehat{LL{R}_{it}}$ as the dependent variable did not produce valuable insights or different results concerning the real estate variables.

### Ex post risk

Several ex post risk measures have been used in the literature. Sinkey and Greenawalt (1991) employed $\frac{net\ charge\ off{s}_{it}}{net\ loans+ charge\ off{s}_{it}}$. Berger and Udell (1990) used loan risk premia as ex ante risk measures while ex post risk was gauged by others through loan charge-offs, overdue 30 days or 30–89 days, and renegotiated. Turning to hypothesis four, we instead follow Delis and Kouretas (2011) who used the ratio of risk assets^9^ to total assets and the ratio of NPLs to gross loans as proxies to evaluate banks’ risk taking. As the authors argued, these measures are better suited to measure banks’ risk taking than a z-score (Mohsni and Otchere 2014), which evaluates the probability of bank insolvency rather than risk engagement. Thus, we use $\frac{impaired\ loan{s}_{it}}{gross\ costumer\ loan{s}_{it}}$ as the variable to describe problem loans. Following the arguments of Salas and Saurina (2002), the resulting variable *impaired*_*it*_ was transformed to $\frac{Impaired\ Loan{s}_{it}}{Gross\ Loan{s}_{it}}/\left(1-\frac{Impaired\ Loan{s}_{it}}{Gross\ Loan{s}_{it}}\right)$.

As Balasubramanyan et al. (2017) pointed out, estimating NPLs using loan loss provisions (LLPs) can be biased, as LLPs may be based on expectations for NPLs, and thus LLPs are not independent from future loan performance. Endogeneity, therefore, has another stake in estimating the equation.^10^ The results of the estimation can be found in Table 6.

**Table 6:** Results of Blundell-Bond-Estimation of ex post appropriateness of loan loss reserves. Ex post risk appropriateness of loan loss reserves is measured by $\frac{loan\ loss\ reserve{s}_{it}}{impaired\ loan{s}_{it}}$. The estimation uses different lag lengths for level and difference instruments and employs Windmeijer’s robust standard errors. Growth variables are denoted by circumflex

	(13)	(14)	(15)	(16)
Dependent variable	LLRIMP_it_	LLRIMP_it_	LLRIMP_it_	LLRIMP_it_
LLRIMP_it − 1_	0.690***	0.688***	0.681***	0. 688***
$\widehat{{GC}{{L}}_{{it}}}$	-176.000	-158.800	-138.200	-54.982
$\widehat{{GC}{{L}}_{{it}-1}}$	-12.000	-10.260	-8.340	-3.415
$\widehat{{GC}{{L}}_{{it}-2}}$	-7.520	-6.735	-5.622	-1.904
TCAR_it − 1_	-0.020	-0.153	0.421	1.428
TCAR_it − 2_	-0.352	-0.444	-0.706	-1.280
TCAR_it − 3_	-0.336	-0.385	-0.396	-0.334
profits_it − 2_	-460.800	-136.200	-368.500	-421.710
profits_it − 3_	870.200	1038.900	780.000	-136.053
Lerner_it_	384.000**	375.000**	304.400*	229.610
Lerner_it − 1_	-94.050	-93.620	-61.590	-67.616
Lerner_it − 2_	-14.040	-14.770	-11.520	-6.227
monitoring_it_	-415.600*	-404.200*	-432.700	-453.596*
monitoring_it − 1_	333.600*	337.500*	358.800	360.116*
monitoring_it − 2_	44.320	33.310	34.050	31.250
CIR_it_	2.246**	2.219**	1.802	1.395
CIR_it − 1_	-0.501	-0.508	-0.336	-0.379
$\widehat{{house}{{P}}_{{it}}}$		-31.950		16.474
$\widehat{{house}{{P}}_{{it}-1}}$		11.540		3.570
$\widehat{{house}{{P}}_{{it}-2}}$		-11.140		-5.734
PRR_it_			0.240	
PRR_it − 1_			-0.042	
PRR_it − 2_			-0.047	
unemp_it_				-2.917
unemp_it − 1_				1.793
unemp_it − 2_				-0.122
N	774	774	774	774
Number of instruments	33	37	37	41
Year dummies	Yes	Yes	Yes	Yes
First order Arellano Bond Test	-2.05**	-2.23**	-1.89*	-2.06**
Second order Arellano Bond Test	0.53	0.52	0.27	0.24
Hansen Statistic	2.31	2.67	2.15	4.74
p value Hansen Statistic	(0.941)	(0.953)	(0.976)	(0.980)

Significance levels are indicated by * *p* < 0.10, ** *p* < 0.05, *** *p* < 0.01

In addition to efficiency, the most obvious finding was the persistence of loan portfolio riskiness, with the lagged dependent variable close to 100%. This variable explained a high share of the variation of the following impaired ratio, rendering at least some of the other lagged variables without individual explanatory power, but rather bundling their effects.

The second finding is that there was a robust positive impact of loan growth on loan risk. Riskier borrowers could be the consequence of the bank already having saturated high quality borrower markets and being forced to lend to bad borrowers due to competitive pressure or bank strategy (closely related to winner’s curse effect). This result is especially meaningful, as we did not find robust effects of loan growth on loan loss provisions, hence the realized losses associated with previous loan volume growth seem to be unanticipated. This impression was reinforced by the even higher coefficient of the lagged growth variable, suggesting weaker predictability of loan losses due to a longer time horizon between loan approval and default.

We expected banking competition to have a positive impact on NPLs, as in Zhang et al. (2018). This is also in line with Herring and Wachter (1999) who argued that disaster myopia could be increased through competition, as non-myopic banks are unable to withstand the pressure that arises if risk premia are too small. This decreases returns and banks increase their leverage. In fact, there is some evidence from our estimations that savings banks with higher market power have lower ex post risk. This result contrasts with the findings of Salas and Saurina (2002), who found that market power increased problem loans for Spanish savings banks.

Finally, current and past house price growth, used as a proxy for expectations on future house price growth, did not appear to influence the ex post risk of savings banks’ loans. Unreported regressions, using $\frac{net\ charge\ off{s}_{it}}{gross\ costumer\ loan{s}_{it}}$ as the dependent variable, as in Sinkey and Greenawalt (1991), confirmed that real estate price growth did not have a significant effect on savings banks’ loan portfolios’ ex post risk. This is line with the results of Koetter and Poghosyan (2010), who found that savings banks were on average less likely to default as a consequence of deviations of housing prices from fundamental values.

### Monitoring, ex post loan loss reserves and deviations from fundamental values

A number of factors can explain the results with regard to ex post risks. As we found that loan growth did not affect risk provisions but realized losses, special attention should be paid to hypothesis 5. The term *ex post loan loss reserves* used in what follows compares loan loss reserves to realized credit risk, i.e. high/low values indicate bad loan loss reserves policy, which could be due to either speculations on rising values of collateral or to overall economic conditions. We tested how real estate price growth affects savings banks’ estimation of risks and their optimism using *LLRIMP*_*it*_, defined as $\frac{loan\ loss\ reserve{s}_{it}}{impaired\ loan{s}_{it}}$.

As loan loss reserves and impaired loans are affected by different lags of the explanatory variables, we included up to three periods of each variable. Again, we estimated a dynamic panel model, as the two components of the dependent variable were endogenous — loan loss reserves and impaired loans were highly persistent. In order to examine the effects of monitoring without including real estate prices, we estimated parsimoniously instrumented models without considering condo price growth as before, but rather estimating a baseline model without real estate variables.

The results in Table 7 suggest that monitoring had an effect on the ratio with varying signs concerning the lags. Current efficiency and market power seemed to have positive impacts on the reserves/losses ratio, which would contradict a too-big-to-fail moral hazard problem.

**Table 7:** Results of Blundell-Bond-Estimation of loan growth with price-to-rent-ratio related explanatory variables. PRR Dummy_it is a dummy variable, indicating whether the price rent ratio in the business area was at least 10 % higher index than the average over all business areas in the respective year. The estimation uses different lag lengths for level and difference instruments and employs Windmeijer’s robust standard errors. Growth variables are denoted by circumflex

	(17)	(18)	(19)	(20)
Dependent variable	$\widehat{{GC}{{L}}_{{it}}}$	$\widehat{{GC}{{L}}_{{it}}}$	$\widehat{{GC}{{L}}_{{it}}}$	$\widehat{{GC}{{L}}_{{it}}}$
$\widehat{{GC}{{L}}_{{it}-1}}$	0.163	0.116	0.205	0.199
impaired_it − 1_	-0.007**	-0.005	-0.008***	-0.006
impaired_it − 2_	0.000	0.000	0.001	0.000
loans/TA_it − 1_	-0.444***	-0.469***	-0.487***	-0.480***
loans/TA_it − 2_	0.059	0.034	0.102	0.119
TCAR_it_	-0.003	-0.003	-0.005**	-0.003
TCAR_it − 1_	0.001	0.001	0.003	0.002
Lerner_it_	-0.141	-0.170	-0.250	-0.192
monitoring_it_	-0.289**	-0.336*	-0.275**	-0.260*
CIR_it_	-0.001	-0.000	-0.001	-0.001
NIM_it_	-0.007	0.010	0.019	-0.009
profits_it − 1_	5.870**	6.394*	5.579*	6.522**
profits_it − 2_	2.464	2.769	1.961	2.203
$\widehat{{{branches}}_{{it}}}$	-0.046**	-0.045**	-0.047**	-0.048**
$\widehat{{{branches}}_{{it}-1}}$	-0.008	-0.011	-0.004	-0.009
PRR Dummy_it_	-0.005			
PRR Dummy_it − 1_	0.001			
${PR}{{R}}_{{it}}^2$		-0.000		
${PR}{{R}}_{{it}-1}^2$		0.000		
Interaction_1_			-0.044	
Interaction_2_			-0.033	
$\widehat{{PR}{{R}}_{{it}}}$				-0.002
$\widehat{{PR}{{R}}_{{it}-1}}$				-0.035
N	546	546	546	546
Number of instruments	72	72	78	72
Year dummies	Yes	Yes	Yes	Yes
First order Arellano Bond Test	-2.96***	-2.84***	-3.18***	-3.11***
Second order Arellano Bond Test	-0.69	-0.65	-0.49	-0.36
Hansen Statistic	48.38	45.03	53.04	45
p value Hansen Statistic	(0.498)	(0.513)	(0.434)	(0.514)

Significance levels are indicated by * *p* < 0.10, ** *p* < 0.05, *** *p* < 0.01

Most importantly, the regression shows that local real estate prices did not induce banks to be over-optimistic. Hypothesis 5 thus is rejected. With regard to the previous estimations, this undermines the finding that savings banks’ loan portfolio risk was not determined directly by real estate prices, but rather by economic factors, which in term helped determine real estate growth. In unreported estimations, we found that the effects of population growth in certain places were even stronger than unemployment rate growth.

As savings banks are backed by public entities and are exposed to less pressure to achieve high gains in the short run, they might unintentionally be less prone to engage in riskier lending. Furthermore, savings banks have strong links with local real estate markets, providing them with local knowledge and enabling them to observe the risks that stem from real estate related lending.

Therefore, besides using price-to-rent-ratios as signal of house price deviations, we calculate fundamental house prices using Pooled-Mean-Group (PMG) estimation, as described by Pesaran et al. (1999) and used by e.g. Kholodilin et al. (2007) and Koetter and Poghosyan (2010) and checked whether savings banks increased their ex post loan loss reserves in response to potential bursting of a housing price bubble. Our model uses population growth, income gauged by gdp per employee and population density as explanatory variables.^11^ The resulting error correction representation of the model therefore is

$$\Delta {{HouseP}}_{{it}}={\phi}_i\left(H{ouseP}_{i,t-1}-{\theta}_0-{\theta}_1 GD{P}_{it}-{\theta}_2{\widehat{population}}_{it}-{\theta}_3 PopDen{s}_{it}\right)+{\lambda}_i\Delta House{P}_{i,t-1}+{\delta}_{1i}\Delta GD{P}_{it}+{\delta}_{2i}\Delta {\widehat{population}}_{it}+{\delta}_{3i}\Delta PopDen{s}_{it}+{\epsilon}_{it}$$

**Fig. 4:**
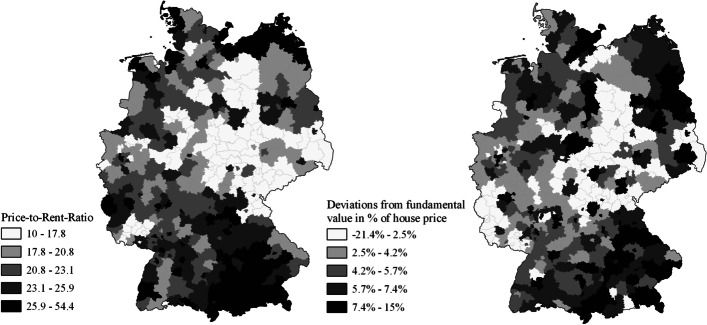
Quintiles of price-to-rent-ratios and deviations from fundamental house prices in % of current house prices in 2017. Data grouped by German fringe counties. Own representation based on data from Empirica AG, own calculations and shape data provided by the German Federal Agency for Cartography

Due to availability, the data for the estimation span from 2005 to 2017. The results are displayed in Table 8. Deviations from fundamental house prices are calculated as $House{P}_{i,t-1}-\hat{\theta_0}-\hat{\theta_1} GD{P}_{it}-\hat{\theta_2}{\widehat{population}}_{it}-\hat{\theta_3} PopDen{s}_{it}$. We additionally plotted the results using German fringe counties to highlight the differences between calculated fundamental real estate prices and price-to-rent-ratios (s. Fig. 4).

**Table 8:** Results of estimation of PMG model of fundamental house prices (Estimation 25).$\widehat{{population}}$ is calculated as percentual growth rate, Population Density as inhabitants per square km (in thsd.) and GDP per Employee is measured in thsd. Euros. Estimation data range from 2005 to 2017

Long run coefficients	
$\widehat{{population}}$	0.290***
	(0.065)
GDP per Employee	0.230***
	(0.014)
Population Density	4.436***
	(0.690)
Short run coefficients	
$\overline{\theta_0}$	-14.431
$\overline{\phi}$	-0.051
$\Delta \widehat{{population}}$	-0.008
ΔGDP per Employee	-0.002
ΔPopulation Density	-0.371
Observations	5083
Log-Likelihood	8559.018

Significance levels are indicated by * *p* < 0.10, ** *p* < 0.05, *** *p* < 0.01

We estimated the impact of this alternative specification of real estate price developments on loan portfolio risk of savings banks using previously used model specifications. The results are displayed in Table 9. As can be seen, as previous measures, deviations from fundamental house prices do not seem to explain savings banks’ loan portfolio risks. Yet, from the results we learn that local variables have a significant impact on house prices. Therefore, impacts of house price developments on loan portfolio risk could be driven by local economy variables in the first place.

**Table 9:** Results of Blundell-Bond-Estimation of Loan growth and loan risk including calculated deviations from fundamental house prices. Instruments and lag lengths are the same as in previous estimations. The estimation uses different lag lengths for level and difference instruments and employs Windmeijer’s robust standard errors. Growth variables are denoted by circumflex

Dependent variable	$\widehat{{{GCL}}_{{it}}}$	*LLR*_*it*_	impaired_it_	LLRIMP_it_
	(26)	(27)	(28)	(29)
$\widehat{{GC}{{L}}_{{it}}}$		-0.602		-169.296
$\widehat{{GC}{{L}}_{{it}-1}}$	0.071	-0.060	0.192*	-13.153
$\widehat{{GC}{{L}}_{{it}-2}}$			0.334**	-8.036
*LLR*_*it* − 1_		0.805***		
impaired_it − 1_	-0.005		0.994***	
impaired_it − 2_	-0.000			
LLRIMP_it − 1_				0.703***
LTA_it_		0.032		
loans/TA_it − 1_	-0.396***			
loans/TA_it − 2_	-0.021			
TCAR_it_	-0.003			
TCAR_it − 1_	0.002	-0.016		0.157
TCAR_it − 2_		0.027	-0.003	-0.385
TCAR_it − 3_			0.009	-0.065
Lerner_it_	-0.063	-0.564		403.807**
Lerner_it − 1_			-1.430*	-105.893
Lerner_it − 2_			0.090	-17.414
monitoring_it_	-0.325**	-0.128		-400.992*
monitoring_it − 1_			-0.591	311.661*
monitoring_it − 2_			0.450	44.729
CIR_it_	0.000	-0.007	-0.000	2.374*
CIR_it − 1_			-0.008**	-0.549
NIM_it_	0.010	0.152		
profits_it − 1_	5.925*	-15.785		
profits_it − 2_	1.026	-3.298		-358.970
profits_it − 3_				438.488
$\widehat{{{branches}}_{{it}}}$	-0.055***			
$\widehat{{{branches}}_{{it}-1}}$	-0.010			
HP Deviation_it_	0.322	1.818		-165.606
HP Deviation_it − 1_	-0.314	-1.673	-0.041	165.133
HP Deviation_it − 2_			0.109	11.730
N	541	1,159	768	761
Number of instruments	71	55	53	36
Year dummies	Yes	Yes	Yes	Yes
First order Arellano Bond Test	-2.89***	-4.17***	-2.00**	-2.04**
Second order Arellano Bond Test	-0.3	0.63	0.99	1.19
Hansen Statistic	48.65	37.54	32.86	2.36
p value	(0.487)	(0.399)	(0.613)	(0.999)

Significance levels are indicated by * *p* < 0.10, ** *p* < 0.05, *** *p* < 0.01

### Panel vector autoregression

As additional check of the robustness of the results, we investigate whether the local economy has a direct impact on loan risk or the former affect house prices which then pass these effects on to loan portfolios. Loan risk hence would be impacted by local economy rather than price increases, thus dismissing explanations via collateral. We estimated a panel vector autoregressive model where we used per capita GDP growth within the business area, unemployment rates, population density as indicator of local urbanization, house price growth, deviations from fundamental house prices and loan growth as endogenous variables besides the mentioned loan portfolio risk variables and, used as additional ex post risk indicator, net charge-offs to average gross loans (*NCO*_*it*_). The results can be found in Table 10. As can be seen, regional variables only have little explanatory power, except for local GDP growth and house price variables, which is surprising, as we would have expected house price growth to have less impact than e.g. unemployment rates. Yet, as GDP has proved to be an important determinant of real estate prices (see Table 8), the effect of real estate price growth could be highly impacted by GDP growth.

**Table 10:** Results of Panel-VAR estimation, robust standard errors in parentheses. *NCO*_*it*_ are banks’ loan net charge offs/average gross loans, *PopDen*_*it*_ is population density within business area, measured as inhabitants/km^2^ and *GDP*_*it*_ is GDP/employee in thsd Euros. Panel specific effects were removed by first differences. Growth variables are denoted by circumflex

	PopDen_it_	unemp_it_	${\widehat{{GDP}}}_{{it}}$	$\widehat{{house}{{P}}_{{it}}}$	HP Deviation_it_	$\widehat{{GC}{{L}}_{{it}}}$	LLR_it_	NCO_it_	impaired_it_	LLRIMP_it_
PopDen_it − 1_	0.679***	-0.001	0.000	0.001***	-0.000	-0.000*	-0.000	-0.000*	-0.000	0.048
	*(0.067)*	*(0.001)*	*(0.000)*	*(0.000)*	*(0.000)*	*(0.000)*	*(0.001)*	*(0.000)*	*(0.001)*	*(0.034)*
unemp_it − 1_	5.898*	1.241***	-0.003	0.041***	-0.008	-0.010	0.006	-0.017*	0.0453	-0.114
	*(3.265)*	*(0.059)*	*(0.004)*	*(0.012)*	*(0.006)*	*(0.009)*	*(0.029)*	*(0.010)*	*(0.032)*	*(1.442)*
${\widehat{{GDP}}}_{{it}-1}$	-171.485***	-6.279***	0.188**	-1.754***	0.888***	-0.901***	0.477*	0.437***	-0.702*	-72.300***
	*(39.761)*	*(0.859)*	*(0.089)*	*(0.236)*	*(0.125)*	*(0.141)*	*(0.272)*	*(0.105)*	*(0.381)*	*(17.633)*
$\widehat{{house}{{P}}_{{it}-1}}$	-188.027***	0.830**	0.011	-0.160	0.018	0.303***	0.568***	0.551***	0.022	-3.315
	*(30.335)*	*(0.405)*	*(0.034)*	*(0.115)*	*(0.050)*	*(0.066)*	*(0.204)*	*(0.085)*	*(0.278)*	*(11.296)*
HP Deviation_it − 1_	64.867	-0.559	0.194	1.393***	0.253	-0.391	-4.091***	-0.765**	-4.221***	2.221
	*(109.710)*	*(1.817)*	*(0.153)*	*(0.478)*	*(0.209)*	*(0.327)*	*(0.955)*	*(0.314)*	*(1.072)*	*(48.140)*
$\widehat{{GC}{{L}}_{{it}-1}}$	-11.649	-1.209**	0.136**	-0.349**	0.186**	-0.408**	0.924**	-0.045**	0.287**	-21.786***
	*(7.255)*	*(0.498)*	*(0.058)*	*(0.139)*	*(0.076)*	*(0.162)*	*(0.385)*	*(0.021)*	*(0.131)*	*(7.806)*
LLR_it − 1_	1.336	-0.056	0.010*	-0.003	0.006	-0.027**	0.783***	0.003	0.119**	-2.599
	*(3.955)*	*(0.071)*	*(0.006)*	*(0.016)*	*(0.008)*	*(0.011)*	*(0.048)*	*(0.012)*	*(0.047)*	*(2.109)*
NCO_it − 1_	-70.635***	1.143***	0.041**	-0.176***	0.005	0.071**	-0.221**	-0.064	0.701***	-9.988
	*(17.211)*	*(0.245)*	*(0.020)*	*(0.062)*	*(0.018)*	*(0.034)*	*(0.112)*	*(0.051)*	*(0.193)*	*(6.209)*
impaired_it − 1_	-27.525***	0.250*	-0.003	0.017	-0.006	0.056**	-0.020	0.061**	0.605***	5.038
	*(9.064)*	*(0.147)*	*(0.012)*	*(0.034)*	*(0.016)*	*(0.022)*	*(0.060)*	*(0.026)*	*(0.099)*	*(4.815)*
LLRIMP_it − 1_	-1.089***	0.011*	-0.001	-0.000	-0.001	0.001	-0.001	0.002***	0.001	0.727***
	*(0.326)*	*(0.006)*	*(0.001)*	*(0.001)*	*(0.001)*	*(0.001)*	*(0.003)*	*(0.001)*	*(0.004)*	*(0.236)*

Significance levels are indicated by * *p* < 0.10, ** *p* < 0.05, *** *p* < 0.01

**Fig. 5:**
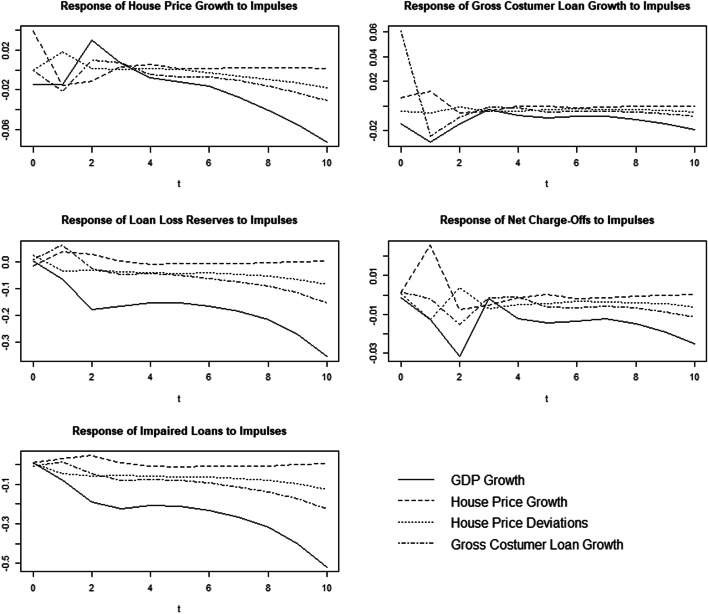
Orthogonalized Impulse Response Functions of selected variables. Besides loan and house price growth we used loan loss reserves as ex ante risk measure and impaired loans to gross costumer loans and net charge-offs to average gross loans as ex post variables

To analyze this relationship closer, we additionally calculated orthogonalized impulse response functions (OIRFs), adopting the procedure described by Sigmund and Ferstl (2021). Therefore, we ordered the local economic variables in the beginning of our estimation as we supposed that effects of local economy are more likely to be passed on to house prices which then affect loan portfolio risk. The results for GDP growth, which proved to have the strongest impact on loan portfolio risk measures, and house price growth and deviations from fundamental values are shown in Fig. 5.

While impulses of house price growth tend to reduce over the course of time, the effects of GDP growth are much more persistent, although on an economically low level. Furthermore, it becomes evident that house price and GDP growth do not move parallel, but in parts even in the opposite direction. The rather pronounced effect of house price growth in the short run is additional evidence that real estate prices could be used as first indicator when making loan decisions, rather than only reflecting local economic figures, which are the basis of loan decisions. Yet, while the effects of real estate growth decline quickly, GDP as underlying factor has a steadier effect on loan portfolio risk measures.

## Conclusions

This paper studies the effects of real estate prices on German savings banks’ risk taking in lending. It contributes to the existing literature in several ways: It analyzes the impact of real estate price growth on loan risk, uses a micro-level perspective to do so, and uses a forward-looking metric to reflect market expectations. In contrast to Koetter and Poghosyan (2010), which is to the best of our knowledge the most similar study that has been published, we do not focus on real estate price deviations from fundamental values on banks’ default probability, but on the effects of real estate price growth on loan portfolio quality. The consequences of a positive impact of real estate price growth on banks’ loan portfolio risk would be severe: On the one hand, local lending would be systematically distorted: While banks lend to risky borrowers in economic prosperous areas with growing real estate prices, banks located in economically lower performing areas with stable or decreasing property prices would act more conservative. Therefore, adverse selection problems in lending obtain a spatial dimension, e.g. when deposits (credits) are shifted to non-risky (risky) banks and thus capital allocation would be impeded.

On the other hand, lending to risky borrowers inflates real estate prices, which in turn leads to additional and even riskier lending. Extensive real estate lending, having a higher loan to asset ratio than other lending (s. Bian and Liu 2018; Blasko and Sinkey 2006) would aggravate risks. Bursting bubbles cannot only result in borrowers’ defaults, but contagion effects of bank defaults could aggravate arising problems quickly. As real estate bubbles frequently are distributed unevenly across spatial entities, micro-evidence on loan portfolio risk and real estate price growth is highly relevant. This not only holds true for German housing and lending markets, but for many countries inheriting banks with politically and geographically limited business.

Overall, there was no robust evidence that real estate price growth has an impact on savings banks’ ex ante or ex post loan portfolio risk. There was only some slight evidence that loan loss reserves were affected by past and current price developments of regional real estate markets, but this effect was dominated by overall economic development. This result is in line with the findings of Koetter and Poghosyan (2010) who find savings banks to be on average less prone to default whereas house price deviations increase banks’ probability of default. Additionally, there was a high persistence of loan factors, which has already been noted as justification for the usage of system GMM. This underscores the relevance of loan maturities when determining NPLs and the significance of collateral in lending. Including those data might offer additional insights into the riskiness of loans collateralized by real estate. Furthermore, as lending practices of savings banks are strongly depending on local economic conditions, which determine real estate prices, there could be some indirect link between loan portfolio risk and real estate markets. Analyzing the impacts of various economic indicators we do not find robust evidence that either local economic conditions have significant impacts on loan portfolio risks.

This result is subject to some limitations with regard to data availability. The observed time span may have been too short to represent a full real estate cycle. The study’s results do not exclude loan losses or the absence of caution by savings banks, as long-term real estate developments were not investigated. Additionally, there has been a steady real increase of real estate prices for virtually all German regions since the onset of the European sovereign debt crisis. Expected loss and loss given default estimates thus still should be monitored thoroughly by banking supervisors.

Another issue is that national economic factors, such as overall economic development or interest rates, have greater explanatory power than regional factors. This may be due to their higher relevance for deposit-lending in the case of interest rates and the consecutive attractiveness of other over-regional business fields like equity investments. As the time dummies were significant in many of the equations, this is a plausible explanation.

The empirical results reject a strong reliance of savings banks on house price growth rates when it comes to lending. This result could not only be the consequence of lending techniques, but also due to legal issues: Banks are legally obliged to consider haircuts in their estimations of LGD (European Parliament, 2013, Capital Requirements Regulations (CRR) I, Art. 181 (1) e))^12^ because borrower risk does not depend on the development of the value of the collateralized real estate (European Parliament, 2013, CRR I, Art. 125 (2) b) and Art. 126 (2) b).

Additionally, due to their geographically-restricted business areas, savings banks are closely connected with their borrowers, allowing them the use of soft information (Berger and Black 2011). While this does not fully replace collateral, local information may help savings banks to correctly forecast borrowers’ future economic conditions and to soften their lending techniques by taking into account factors other than collateral for their loan decisions.

Future developments in business administration and management might thus be crucial for local banks and their environment: Shifting their techniques from relationship to transaction-based lending could not only decrease costumer relationships and induce banks to engage in higher risks, but also reduce the availability of funding in regions with weaker economic performance. The economic consequences of the ongoing pandemic-induced de-personalization of interaction and business could thus have long lasting effects on underperforming areas and widen interregional economic gaps.

## Appendix: Lerner index

We briefly present choice and calculation of the measure of competition using the Lerner Index. For locally based savings banks, measures for competition that take into account several dimensions of local lending and borrowing include local wealth, share of county deposits, number of branches within an area, interest income per branch, etc. Yet, most of this data was either not available at all or had low explanatory power due to a variety of factors, such as different hierarchies of branches of rival commercial banks. Furthermore, classical measures of market concentration, such as Herfindahl-Indices on deposits on a county level, are very sensitive to the non-homogeneity of banks (Forssbaeck and Shehzad 2015). Additionally, variables are commonly not bank-specific, but rather locally dependent, and their effects can be gauged by county-dummies.

Thus, as a common index on a bank-level, we employ a Lerner-Index based on the procedure described in Berger et al. (2009) and Feldkircher and Sigmund (2017). The Lerner index is defined as

$$Lerne{r}_{it}=\frac{Price\ of\ Total\ Asset{s}_{it}- Marginal\ Cost\ of\ Total\ Asset{s}_{it}}{Price\ of\ Total\ Asset{s}_{it}}$$

Where marginal costs are derived from

$$Marginal\ Cost\ of\ Total\ Asset{s}_{it}=\frac{Cos{t}_{it}}{TA_{it}}\left[{\beta}_0+{\beta}_1\ln \left({TA}_{it}\right)+\sum\limits_{k=1}^3{\phi}_k{\ln}{F}_{k, it}\right]$$

With costs stemming from the translog function:

$$\displaystyle{ \begin{array}{c}\ln Cos{t}_{it}={\beta}_0+{\beta}_1\ln \left({TA}_{it}\right)+{\beta}_2\frac{1}{2}{\left(\ln \left(T{A}_{it}\right)\right)}^2+\sum\limits_{k=1}^3{\gamma}_k{\ln}{F}_{k, it}+\sum\limits_{k=1}^3{\phi}_k\ln \left(T{A}_{it}\right){\ln}{F}_{k, it}\\ {}+{\alpha}_1\ln \left({F}_1\right)\cdot \ln \left({F}_2\right)+{\alpha}_2\ln \left({F}_1\right)\cdot {\ln}\left({F}_3\right)+{\alpha}_3\ln \left({F}_2\right)\cdot \ln \left({F}_3\right)+\sum\limits_{k=1}^3{\alpha}_{3+k}{\ln}{F}_{k, it}^2\end{array}}$$

Total assets are described by *TA*_*it*_, input costs by *F*_*k*_ : *F*_1_ is labor costs, described by $\frac{staff\ expenses}{TA}$, *F*_2_ are costs of funds ($\frac{interest\ expense}{total\ deposits}$) and *F*_3_ are costs of fixed capital ($\frac{operating\ expenses}{TA}$). The dependent variable (total costs) is calculated as the sum of total operating expenses and total interest expenses.

The results of the estimation of the equation above can be found in Table 11.

**Table 11:** Results of estimation of translog cost function in order to calculate the Lerner Index for each bank. Estimations were conducted using year fixed effects and clustered standard errors. Dependent variable is the natural logarithm of total operative expenses and interest expenses in Euro

ln(Total Assets)	1.261***
	(17.120)
0.5 [ln (Total Assets)]^2^	-0.018***
	(-3.350)
F1	0.294*
	(2.070)
F2	-0.236***
	(-4.850)
F3	1.365***
	(8.630)
ln(Total Assets)·F1	-0.021**
	(-2.720)
ln(Total Assets)·F2	0.012***
	(4.270)
ln(Total Assets)·F3	-0.023**
	(-3.250)
F1·F2	0.002
	(0.350)
F1·F3	-0.016
	(-1.500)
F2·F3	-0.036**
	(-3.270)
F1·F1	-0.007
	(-1.770)
F2·F2	0.000
	(0.100)
F3·F3	0.053***
	(6.270)
Constant	-1.914***
	(-3.510)
Observations	8,672
R^2^ (overall)	0.992

Significance levels are indicated by * *p* < 0.10, ** *p* < 0.05, *** *p* < 0.01

As in Berger et al. (2009), we used year fixed effects and robust standard errors (clusters on a bank-level basis). At first glance, there were two issues that would reject the use of year dummies. First, F-Tests suggested that the year dummies were jointly insignificant for some specifications. Second, they captured overall economic conditions, which are supposedly of less relevance for regionally non-systemic banks. They were maintained in all estimations, however, as the effects of (national) interest levels and other economic conditions would otherwise be falsely assigned to local house price growth. Many of the coefficients’ values and significances were similar to the results of Feldkircher and Sigmund (2017).^13^

The obtained coefficients were then used together with the input data described above to calculate marginal costs, which are then used to calculate each bank’ s Lerner-Index.

## Footnotes

1 See also Berger and Udell (1995) who find that collateralized loans are riskier than unsecured, with the overall risk of the former being reduced by their collateral.

2 Further analysis of substitution of monitoring by collateral can be found in Niinimäki (2009).

3 Salas and Saurina (2002) mention the possibility that savings banks could have higher ex post risks due to financing riskier projects in favor of local economy.

4 The finding of Christians and Gärtner  (2014) that German savings banks in rural areas demand higher collateral from their borrowers support the theoretical predictions of Inderst and Mueller (2006).

5 As current earnings can be related to the risk level of bank lending, lagged values are used (Delis and Kouretas 2011).

6 Note that a mere substitution of monitoring by increasing real estate prices should not alter risk taking, thus a separation between effects is to be expected.

7 Contrarily, Cyree and Morris (2018) find that banks in high income and low population counties mostly outperform those located in low income counties. Thus, local economic factors seem to matter or at least attract more/less talented managers.

8 Inclusion of additional lags did not produce additional significant coefficients.

9 Under risk assets the authors subsume all assets with volatile values and lead to varying profits (Delis and Kouretas 2011).

10 Balasubramanyan et al. (2017) use a 2SLS procedure to overcome this problem by first estimating LLP using current NPLs and using the estimates when quantifying NPL.

11 Due to the rather short longitude of the data, we used one lag only (s. Koetter and Poghosyan 2010). Specifications using more lags did not result in superior models.

12 s. Besanko and Thakor (1987)
